# Diagnostic Value of Combined Intravoxel Incoherent Motion Diffusion-Weighted Magnetic Resonance Imaging with Diffusion Tensor Imaging in Predicting Parametrial Infiltration in Cervical Cancer

**DOI:** 10.1155/2021/6651070

**Published:** 2021-05-11

**Authors:** Ting-Ting Lin, Xin-Xiang Li, Wei-Fu Lv, Jiang-Ning Dong, Chao Wei, Ting-Ting Wang, Chuan-Bin Wang, Ping Zhang

**Affiliations:** ^1^Anhui Provincial Hospital, Cheeloo College of Medicine, Shandong University, Jinan, Shandong 250012, China; ^2^Department of Radiology, The First Affiliated Hospital of USTC, Division of Life Sciences and Medicine, University of Science and Technology of China, Hefei, Anhui 230031, China; ^3^Anhui Provincial Hospital, Department of Radiology, The First Affiliated Hospital of USTC, Division of Life Sciences and Medicine, University of Science and Technology of China, Hefei, Anhui 230001, China

## Abstract

**Objective:**

This study sought to determine the diagnostic value of combined intravoxel incoherent motion (IVIM) diffusion-weighted magnetic resonance imaging (MRI) with diffusion tensor imaging (DTI) in predicting parametrial infiltration (PMI) in patients with cervical cancer.

**Materials and Methods:**

We enrolled 65 patients with cervical cancer confirmed by radical hysterectomy (25 PMI-negative and 40 PMI-positive) who underwent IVIM and DTI pretreatment. The parameters of IVIM (ADC, D, D ^*∗*^, and f) and DTI (average diffusion coefficient (DCavg) and fractional anisotropy (FA)) were recorded by two observers. All parameter differences were tested, and the receiver operating characteristic (ROC) curves were generated to estimate the diagnostic performance of significant metrics and their combinations.

**Results:**

Compared to the PMI-negative group, the PMI-positive group had significantly lower D (0.632 ± 0.017 *vs.* 0.773 ± 0.024, *p* < 0.001) and lower FA (0.073 ± 0.002 *vs.* 0.085 ± 0.003, *p*=0.003). The area under the ROC curve (AUC) of D and FA was 0.801 and 0.726, respectively, and the combination of D and FA improved the AUC to 0.931, with a sensitivity and specificity of 80.0% and 97.5%, respectively.

**Conclusion:**

D and FA values could be used to help diagnose PMI in patients with cervical cancer. The combination of IVIM and DTI was more valuable than either option alone.

## 1. Introduction

Cervical cancer is the third most prevalent type of malignant tumor in females worldwide. Parametrial infiltration (PMI) is a key predictor of a poor prognosis and is associated with increased rates of tumor metastasis, tumor recurrence, and mortality [1–4]. Accurate pretreatment evaluation of PMI is needed to determine the International Federation of Gynecology and Obstetrics (FIGO) stage of the cervical cancer and select individualized treatment strategies [5]. Magnetic resonance imaging (MRI), as an accurate and noninvasive pretreatment method, has immense clinical value in evaluating PMI.

MRI techniques have been used in several studies to assess PMI in patients with cervical cancer. When considering tumor volume combined with findings that are suggestive of PMI, preoperative MRI is useful in ruling out PMI diagnosis [6]. As shown by Park et al., the apparent diffusion coefficient (ADC) of cervical cancer is lower in PMI-positive than PMI-negative cases [4, 7]. Woo et al. showed that 3.0 T MRI scanners and diffusion-weighted imaging (DWI) can be used to improve the diagnostic efficiency of detecting cervical cancer with PMI [8]. Intravoxel incoherent motion (IVIM) is the stretched modality of conventional DWI sequence based on a bi-exponential model and the derived parameters include standard ADC (ADC), slow ADC (D), fast ADC (D ^*∗*^), and perfusion fraction (*f*). The ADC value quantifies the pure water molecular diffusion (represented by D) and microcirculation perfusion (represented by D ^*∗*^). All parameters can be measured simultaneously, which can better reveal the microstructure of tumors and biological issues and has been used for diagnosing, differentiating pathological subtype and histological grade, and evaluating treatment efficacy of cervical cancer [9–11].

In addition, diffusion tensor imaging (DTI) is widely used in the study of uterine diseases. In 2006, Weiss et al. investigated the use of 3-dimensional (3D) imaging of normal fiber architecture of an *ex vivo* human uterus by DTI [12]. In another *ex vivo* study, Toba et al. found that DTI is a beneficial and complementary technique in detecting myometrial invasion in patients with endometrial cancer [13]. In an *in vivo* investigation, He et al. showed that the dynamic changes of fractional anisotropy (FA) are correlated with menstrual cycles [14]. Another *in vivo* study by Fujimoto et al. compared DTI parameter variations in different uterine layers during the luteal phase of menstrual cycles [15]. DCavg and FA are the main quantitative parameters obtained from DTI data [16]. DCavg value reflected water diffusion in all directions and FA value represented the proportion of the anisotropic components of the water molecule to the overall diffusion tensor. The quantitative parameters have been shown to be useful in detecting, diagnosing, and predicting pathological grade of tumors [17, 18].

To the best of our knowledge, the method of combining IVIM and DTI to detect PMI in cervical cancer cases has not been published previously. The purpose of the current study was, therefore, to investigate the diagnostic value of the combination of IVIM and DTI for predicting PMI in cervical cancer.

## 2. Materials and Methods

### 2.1. Patients

This study was supported by our institutional review board, and all patients provided written informed consent to participate. Between February 2019 and April 2020, information from 90 consecutive patients with clinically suspected or pathologically proven cervical cancer was retrieved from our hospital. This study's inclusion criteria were as follows: (1) patients had cervical cancer diagnosed by pathology, (2) patients had not undergone biopsy, surgery, or other treatment, before MRI evaluation, (3) conventional MRI was performed with uniform IVIM, with DTI protocols performed simultaneously before treatment, and (4) the image quality was adequate for subsequent analysis (no obvious artifacts and serious imaging distortion; the maximum diameter of tumor was more than 1 cm and displayed clearly). The exclusion criteria were as follows: (1) the lesion diameter was too small to measure (<1 cm), (2) the MRI scan sequences were incomplete, or (3) the image quality could not meet the needs of diagnosis and measurement (imaging with obvious motion artifacts or distortion).

### 2.2. MRI Evaluation

MRI data were acquired using a 3.0T scanner (Signa HDxt; GE Healthcare, Milwaukee, Wisconsin, USA) with an 8-channel phased array body coil. All patients received an intramuscular injection of 15 mg of scopolamine butyl bromide 30 minutes before the examination to reduce artifacts caused by intestinal movement. Pelvic imaging included routine sequences (axial T1-weighted imaging (T1WI), axial/sagittal T2-weighted imaging (T2WI), and axial T2WI with fat suppression), functional imaging (IVIM and DTI), and contrast-enhanced (CE) protocols. CE-MRI was performed after an intravenous injection of gadodiamide (0.1 mmol/kg, 3 ml/s; Omniscan, GE Healthcare, Cork, Ireland). All scanning processes were performed with free breathing.

IVIM with 10*b* values (0, 10, 20, 50, 100, 200, 400, 800, 1000, and 1200 s/mm^2^) was obtained with single-shot spin-echo echo planar imaging (repetition time (TR) = 4000 ms, echo time (TE) = 85 ms, slice thickness = 4 mm, number of excitations (NEX) = 2, matrix size = 130 × 128, and field of view (FOV) = 40 × 22 cm). The DTI parameters were TR = 7000 ms, TE = 89.6 ms, slice thickness = 4 mm, diffusion direction = 15, number of excitations (NEX) = 1, matrix size = 96 × 130, and FOV = 32 cm. The acquisition time for IVIM and DTI was 5 min, 44 s, and 1 min, 59 s, respectively.

### 2.3. Radiological Evaluation

The original IVIM and DTI data were transferred to the Advantage Windows 4.5 workstation and processed using the in-house Function Tool (GE Healthcare, Milwaukee, Wisconsin, USA). The signal-to-noise ratio (SNR) of the images was evaluated before analysis. The MADC or Diffusion Tensor interface was chosen for IVIM or DTI data set respectively after entered the Function Tool software. Subsequently, the IVIM parameters (ADC, D, D ^*∗*^, and *f*) and DTI metric maps (DCavg and FA) were generated automatically. All data were evaluated by 2 radiologists with 21 (observer 1) and 15 (observer 2) years of experience in gynecological imaging. The two radiologists were blinded to measure with each other's findings. The IVIM dataset was processed using the bi-exponential model fitting formula [19]: *Sb*/*S*0 = (1 − *f*) ^*∗*^ exp (−*b* ^*∗*^D) + *f* ^*∗*^ exp [*b* ^*∗*^ (D ^*∗*^ + D)], where *b* was the *b*-value represents the diffusion sensitizing factors, D indicated the slow component of diffusion, D ^*∗*^ stood for the incoherent microcirculation, *f* was the fractional perfusion linked to the intravascular component, *Sb* reflects the mean signal intensity with the diffusion gradient *b*, and *S*0 reflects a 0 of *b* value. Referencing the T2WI and CE T1WI images, the maximum solid area of the tumor with the most restricted diffusion and the most obvious display of IVIM images and original DTI images was outlined as the regions of interest (ROIs). ROIs were drawn manually as large as possible along the tumor margin of the tumor's largest area. Macroscopic cystic degeneration, necrotic regions, hemorrhage, surrounding vessels, and artifacts induced by air-water interface were avoided during the tumor contour. Each tumor was measured 3 times in the selected ROI regions on IVIM and DTI parametric maps. Measurements were performed by observer 1 twice, the interval between the 2 measurements being at least 1 week. The third measurement was performed by observer 2. The arithmetic mean of the 3 measurements by the 2 observers was recorded as the final result and was used for the following statistical analysis.

### 2.4. Statistical Analysis

Statistical analyses were conducted with SPSS software version 23.0 (Chicago, IL, USA) and GraphPad Prism 8.0 (San Diego, CA, USA). A *p* value <0.05 was considered to indicate statistical significance. An intraclass correlation coefficient (ICC) was calculated to test intraobserver (using the 2 results obtained by observer 1) and interobserver (using the first result from observer 1 and the single result by observer 2) reproducibility (an ICC value > 0.8 representing an almost perfect agreement). Quantitative data were described as mean ± standard deviation (SD). A one-way analysis of variance was used to estimate the data with a normal distribution; the comparison of parameters between two groups was performed using least significant difference (LSD) test. Mann–Whitney test in the nonparametric test was used to compare the data with a nonnormal distribution. Receiver operating characteristic (ROC) curves were calculated to evaluate the diagnostic performance of the significant parameters and their combinations. The optimal cut-off value of each parameter was determined by the maximum of Youden index (the maximum of Youden index = sensitivity + specificity − 1).

## 3. Results

### 3.1. Patients

A total of 65 patients between the ages of 27 and 88 years (median age: 53.0 years) with pathologically confirmed cervical cancer were enrolled in the study. The staging was made according to the FIGO staging criteria of 2018 [20]. The final diagnosis revealed that 40/65 (61.5%) of the patients had low-FIGO-stage cervical cancer (IB to IIA) without PMI and 25/65 (38.5%) had high-FIGO-stage cervical cancer (IIB to IVA) with PMI. The clinical 2018 FIGO stage breakdown was IB, *n* = 27; IIA, *n* = 13; IIB, *n* = 11; IIIA, *n* = 7; IIIB, *n* = 4; and IIIC, *n* = 3.

### 3.2. Reproducibility of IVIM and DTI Measurements

The intraobserver ICC, obtained using the 2 measurements taken by observer 1, ranged from 0.867 to 0.925 (Table 1). The interobserver ICC, obtained using observer 1's first result and observer 2's single result, ranged from 0.836 to 0.934 (Table 1). Both the intra- and interobserver assessments indicated excellent reproducibility.

### 3.3. Diagnostic Efficiency of IVIM Parameters, DTI Metrics, and Their Combination

**Figure 1:**
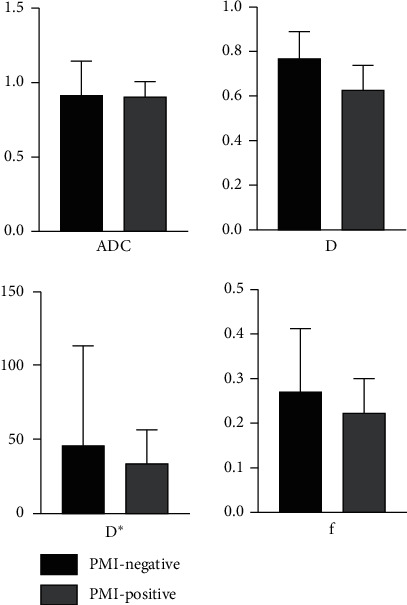
ADC, D, D ^*∗*^, and *f* in the PMI-positive and PMI-negative groups.

**Figure 2:**
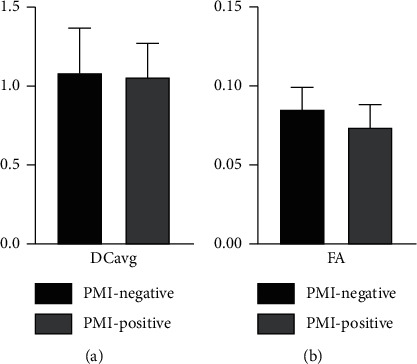
DCavg and FA in the PMI-positive and PMI-negative groups.

IVIM-, DWI-, and DTI-derived parameters between the PMI-positive and PMI-negative groups were compared and are summarized in Table 1 and Figures 1 and 2. Compared to the PMI-negative group, the PMI-positive group demonstrated significantly lower D (0.632 ± 0.017 *vs.* 0.773 ± 0.024, *p* < 0.001) and lower FA (0.073 ± 0.002 *vs.* 0.085 ± 0.003, *p* < 0.05); however, there was no significant difference for ADC, D ^*∗*^, *f*, and DCavg.

**Figure 3:**
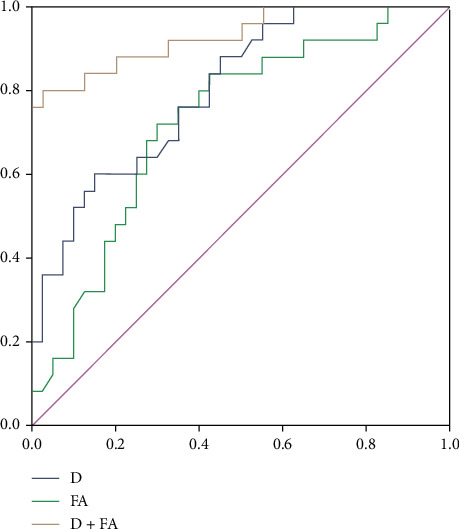
ROC curve showing D (blue line), FA (green line), and the combination of D and FA (yellow line) in the diagnosis of cervical cancer with parametrial invasion.

**Figure 4:**
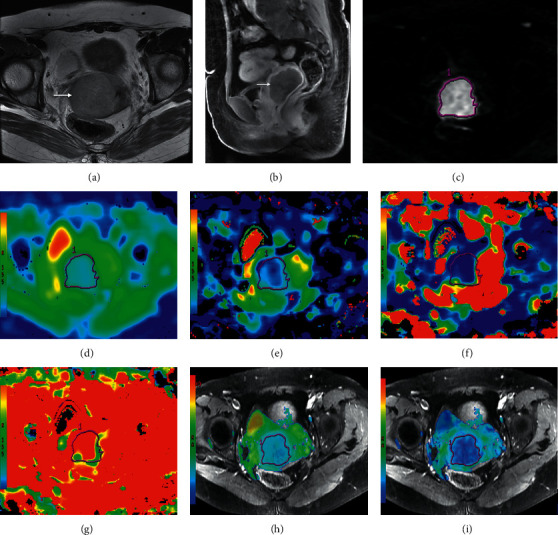
A 52-year-old woman with stage IIA cervical cancer. (a) is T2-weighted image (T2WI) shows a slightly hyperintense cervical mass, which was slightly enhanced on the contrast-enhanced magnetic resonance image (b). (c) shows the tumor with hypertension on the IVIM-DWI image with the *b* value of 800 s/mm^2^. (d–g) display the ADC, D, D ^*∗*^, and *f* parametric maps are derived from IVIM-DWI. (h–i) show the DCavg and FA maps derived from DTI.

ROC analyses revealed that setting the cut-off value of D to 0.741 × 10^−3^ mm^2^/s resulted in the diagnostic efficiency for differentiating between PMI-positive and PMI-negative lesions, with a sensitivity of 60.0% and a specificity of 85.0% (AUC = 0.801) (Table 2). When setting the cut-off value of FA to 0.078, differentiation between PMI-positive and PMI-negative lesions achieved a sensitivity of 72.0% and a specificity of 70.0% (AUC = 0.726). The combination of D and FA improved the diagnostic performance obviously, with the highest AUC (0.931), sensitivity (80.0%), and specificity (97.5%) (Figure 3). Representative IVIM-DWI and DTI images of the patient are shown in Figure 4.

## 4. Discussion

This study investigated IVIM and DTI in cervical cancer with PMI in 65 patients. To the best of our knowledge, this is the first study to use these technologies to evaluate PMI in patients with cervical cancer. Moreover, we found the D and FA value showed a significant difference between the PMI-positive and PMI-negative groups, especially the combination of D and FA improving the diagnosis of PMI in cervical cancer.

IVIM-DWI with multiple *b* values using a double exponential model reflects the microcirculatory and diffusivity information of tumors [21, 22]. There were no differences between the PMI-positive and PMI-negative groups in terms of ADC, D ^*∗*^, and *f* values in this study, which is reflective of differences in tumor heterogeneity, tissue cellularity density, and tissue perfusion. The ADC value quantifies tissue water molecule diffusion and reflects the capillary microcirculation perfusion [23]. The D ^*∗*^ value represents the microcirculation perfusion and tissue microstructure in cervical cancers, and the *f* value represents the volume ratio of the local microcirculation perfusion and the overall effect of diffusion [11, 24]. The lack of significant differences between the PMI-positive and PMI-negative groups indicate that they were the similar in terms of water molecule diffusion and angiogenesis. In our study, there was a significant difference in the D value of the PMI-positive and PMI-negative groups. Perucho et al. [25] showed that high FIGO stages had higher D ^*∗*^ values than low stages, but D values had no significance in distinguishing between high and low stages. The D value, excluding the low *b* value perfusion, represents pure water molecule diffusion of the tumor. The diffusion restriction of the tumor is related to cell gap. In this study, the D value of the PMI-positive group was smaller than that of the PMI-negative group, which means that the tumor cell gap in the PMI-positive group was smaller than that in the PMI-negative group. This feature reflects that the tumor cells with indefinite direction growth in the PMI-positive group were more obvious than those of the PMI-negative group, which indirectly reflects the higher degree of malignancy of the PMI-positive group.

DCavg values are affected by cell density, cell organization, and microcirculation [15, 26]. In our study, there was no significant difference between the PMI-positive and PMI-negative groups, indicating that the DCavg value could not be used to diagnose PMI in cervical cancer cases. The FA value is mainly related to cell density, the form of nucleus division, and the arrangement of muscle fibers [27]. The present study showed that compared with the PMI-negative group, the PMI-positive group had a lower FA value. This result corresponded with a previous report that the FA values of endometrial cancer (0.21 ± 0.05) were lower than those of the inner myometrium (0.44 ± 0.01) and outer myometrium (0.32 ± 0.08), while the DCavg values of endometrial cancer could not be used to evaluate myometrial invasion [13]. The tumor cells infiltrated into the space of muscle fiber bundles and disrupted a varying degree of muscle fibers, resulting in a decreased amount of muscle fiber bundles in the unit voxel. After that, the water diffusive limitation in all directions increased. In our study, the PMI-positive group showed a higher degree of malignancy than the PMI-negative group, reflecting higher damage to the muscle fibers. Meanwhile, the space of the muscle fibers decreased.

Using a combination of D and FA resulted in the best diagnostic performance in terms of diagnosing PMI, and use of the combination was significantly better than the use of D or FA alone. Moreover, the combination of D and FA had higher sensitivity and specificity (80.0% and 97.5%, respectively) than either alone. Therefore, a combination of IVIM and DTI could be used to diagnose PMI with improved efficacy. This improvement might considerably benefit clinical decision-making, facilitate the management of individual patients, and improve patients' clinical prognosis.

Several limitations of the present study should be mentioned. First, the number of patients with cervical cancer was relatively small. Second, a wide range of clinical stages (early and advanced cervical cancer) were included in the study, and different pathological subtypes were not analyzed. Third, certain biases related to this study were inevitable because none of our team members were involved in the initial diagnostic processes or documentation of treatment. In addition, the ROIs were drawn manually by radiologists in the study, so certain subjective factors of inter- and intraobserver variability and slice-selection bias may exist. Future research with a larger sample size will be carried out.

## 5. Conclusions

The present study showed that PMI-positive lesions had significantly lower D and lower FA values than PMI-negative lesions. Both IVIM and DTI were promising methods for diagnosing PMI in cervical cancer cases. However, the combination of IVIM and DTI is more valuable than either option alone, as the combination obviously improved the diagnostic performance, resulting in higher AUC and higher diagnostic sensitivity and specificity than either option alone.

## Figures and Tables

**Table 1:** Comparison of IVIM-, MRI-, and DTI-derived parameters between the PMI-positive and PMI-negative groups.

Parameters	PMI-negative (*n* = 40)	PMI-positive (*n* = 25)	*P* value	ICC	
				Inter (95% CI)	Intra (95% CI)
IVIM-MRI					
ADC	0.918 ± 0.045	0.904 ± 0.016	0.732	0.932 (0.865, 0.973)	0.867 (0.789, 0.943)
D	0.773 ± 0.024	0.632 ± 0.017	＜0.001	0.934 (0.869, 0.958)	0.902 (0.833, 0.972)
D ^*∗*^	46.22 ± 13.46	33.90 ± 3.63	0.290	0.865 (0.796, 0.924)	0.906 (0.858, 0.965)
f	0.273 ± 0.028	0.225 ± 0.076	0.080	0.932 (0.862, 0.974)	0.867 (0.784, 0.921)

DTI					
DCavg	1.080 ± 0.058	1.062 ± 0.033	0.765	0.925 (0.834, 0.946)	0.912 (0.872, 0.978)
FA	0.085 ± 0.003	0.073 ± 0.002	0.003	0.836 (0.778, 0.895)	0.925 (0.876, 0.954)

**Table 2:** Diagnostic efficiency of each metric and their combined parameters.

Parameters	Cut-off value	AUC	Sensitivity	Specificity
D	0.741	0.801 (0.695–0.907)	0.600	0.850
FA	0.078	0.726 (0.600–0.852)	0.720	0.700
D + FA	—	0.931 (0.865–0.997)	0.800	0.975

## Data Availability

The data used to support the findings of this study are included within the article.
